# Supplementary material for: Shedding Light on Heavy Metal Contamination: Fluorescein-Based Chemosensor for Selective Detection of Hg2+ in Water
Source: Int J Mol Sci. 2024 Mar 10;25(6):3186. doi: 10.3390/ijms25063186 (PMC10970617; doi:10.3390/ijms25063186)
Supplement: Supplementary file 1 [file ijms-25-03186-s001.zip › ijms-2899313-supplementary.pdf]

Supporting Information

Shedding Light on Heavy Metal Contamination: Fluorescein-Based Chemosensor for Selective Detection of Hg^{2+} in Water

Maksim N. Zavalishin [1,*], Alexey N. Kiselev [2], Alexandra K. Isagulieva [3,4], Anna V. Shibaeva [5], Vladimir A. Kuzmin [5,6], Vladimir N. Morozov [5], Eugene A. Zevakin [7], Ulyana A. Petrova [1], Alina A. Knyazeva [1], Alexey V. Eroshin [1], Yuriy A. Zhabanov [1] and George A. Gamov [1]

[1] Faculty of Inorganic Chemistry and Technology, Ivanovo State University of Chemistry and Technology, 153000 Ivanovo, Russia; petrowa.ulya2015@yandex.ru (U.A.P.); knyazeva_alina1@mail.ru (A.A.K.); alexey.yeroshin@gmail.com (A.V.E.); zhabanov@gmail.com (Y.A.Z.); ggamov@isuct.ru (G.A.G.)

[2] G.A. Krestov Institute of Solution Chemistry, Russian Academy of Sciences, 153045 Ivanovo, Russia; scatol@yandex.ru

[3] Burnazyan Federal Medical Biophysical Center, Federal Medical Biological Agency of the Russian Federtion, 123182 Moscow, Russia; kia2303@ya.ru

[4] Institute of Gene Biology, Russian Academy of Sciences, 119991 Moscow, Russia

[5] Emanuel Institute of Biochemical Physics, Russian Academy of Sciences, 119334 Moscow, Russia; anna-shiba@mail.ru (A.V.S.); vladimirkuzmin7@gmail.com (V.A.K.); morozov.v.n@mail.ru (V.N.M.)

[6] National Research Nuclear University MEPhI, 115409 Moscow, Russia

[7] Vernadsky Institute of Geochemistry and Analytical Chemistry, Russian Academy of Sciences, 119991 Moscow, Russia; ezevakin.zev@yandex.ru

* Correspondence: zavalishin_mn@isuct.ru; Tel.: +7-9106957233

Table of Contents

1. **Figure S1.** Results of predicting the sensing ability of chemosensor **1** towards metal ions based on chemical formulae
2. **Figure S2.** MALDI TOF mass spectrum of mixture of chemosensor **1** + Hg^{2+}
3. **Figure S3.** Example of titration results of chemosensor 1 with Hg^{2+} ions in KEV software (stoichiometric model M:L = 1:1)
4. **Figure S4.** Example of titration results of chemosensor 1 with Hg^{2+} ions in KEV software (stoichiometric model M:L = 1:2)
5. **Figure S5.** Possible reaction scheme between chemosensor **1** and Hg^{2+} ion
6. **Figure S6.** The fluorescence detection limit of chemosensor **1** in H_2O-DMSO (8:2 v:v)
7. **Table S1.** Structures of selected fluorescence probes for Hg^{2+} ions in the literature
8. **Figure S7.** An example of spectral measurements of chemosensor **1** in the presence of Hg^{2+} ions (30 μM) in the sample (Uhtokhma river)
9. **Figure S8.** Two conformers of **1** and transition state molecular models
10. **Table S2.** Calculated composition of the lowest excited states and corresponding oscillator strengths for **1**
11. **Table S3.** Shapes of molecular otbitals participating in electronic transitions

12. **Figure S9.** IR spectrum of chemosensor **1**
13. **Figure S10.** MALDI TOF mass of chemosensor **1**
14. **Figure S11.** ^{1}H NMR spectra of chemosensor **1**
15. **Figure S12.** ^{13}C NMR spectra of chemosensor **1**
16. **Figure S13.** Cartesian coordinates of quantum chemical structures

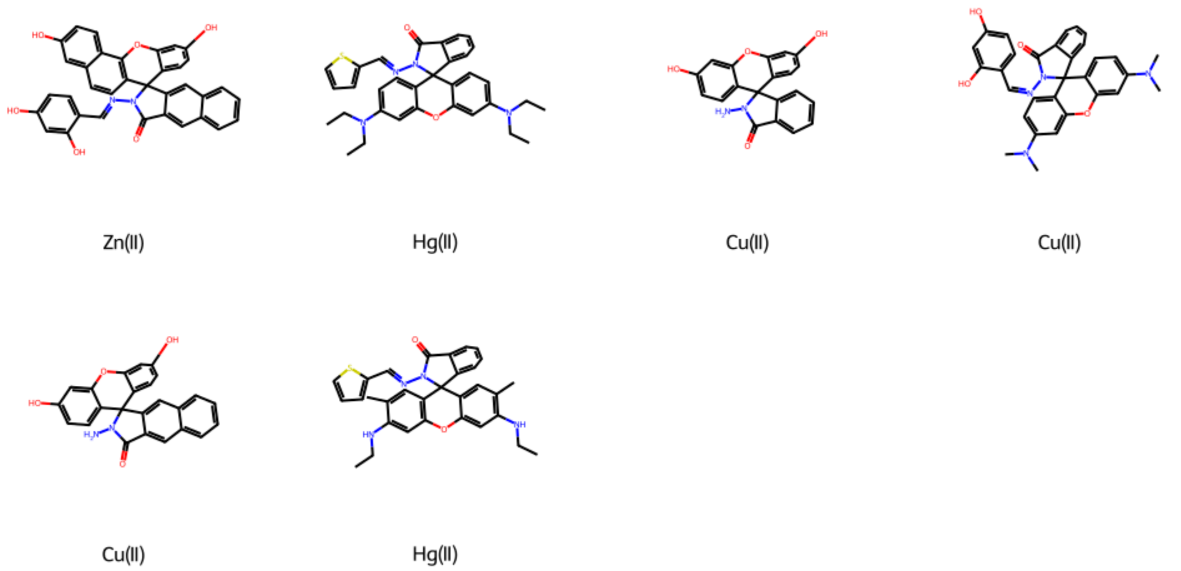

Figure S1. Results of predicting the sensing ability of chemosensor **1** towards metal ions based on chemical formulae

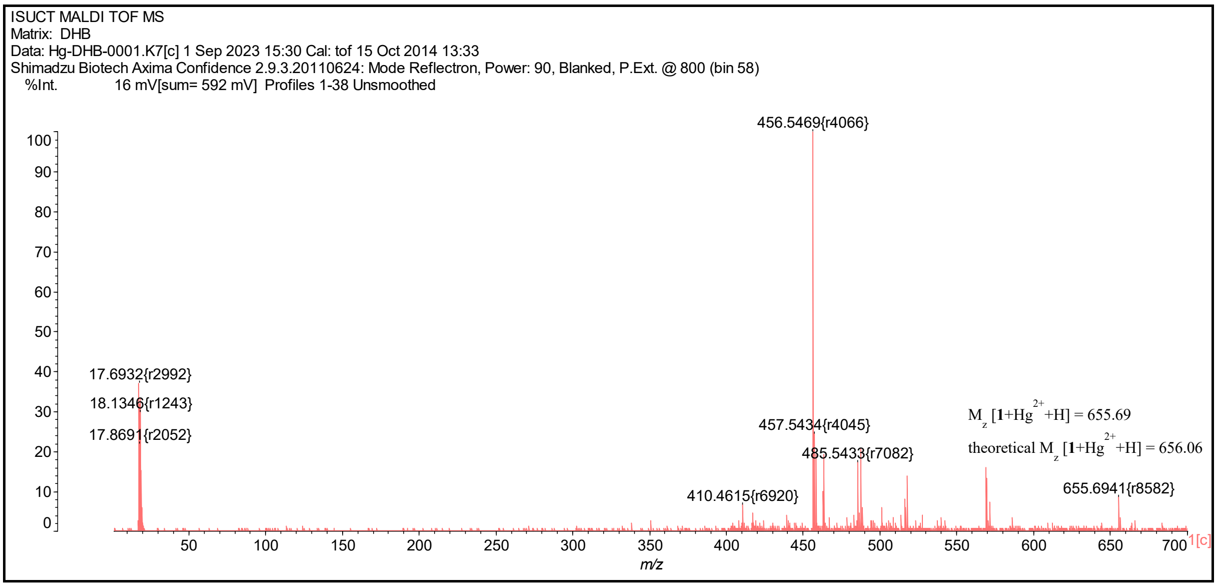

Figure S2. MALDI TOF mass spectrum of mixture of chemosensor **1** + Hg^{2+}

Evaluated Constants

	Component	Constant	St.Deviation	Validity
1	M	0	0	OK
2	L	0	0	OK
3	ML	3.54354858398438	0.242498103256484	OK

csv xlsx

Correlation Matrix

	ML
ML	1

csv xlsx

Adjusted R^2

	Adj. R^2
1	0.969502

csv xlsx

Figure S3. Example of titration results of chemosensor 1 with Hg^{2+} ions in KEV software (stoichiometric model M:L = 1:1)

Evaluated Constants

	Component	Constant	St.Deviation	Validity
1	L	0	0	OK
2	M	0	0	OK
3	ML	-17.5	230800787.724287	Insignificant

csv xlsx

Correlation Matrix

	ML
ML	1

csv xlsx

Adjusted R^2

	Adj. R^2
1	0.964247

csv xlsx

Figure S4. Example of titration results of chemosensor 1 with Hg^{2+} ions in KEV software (stoichiometric model M:L = 1:2)

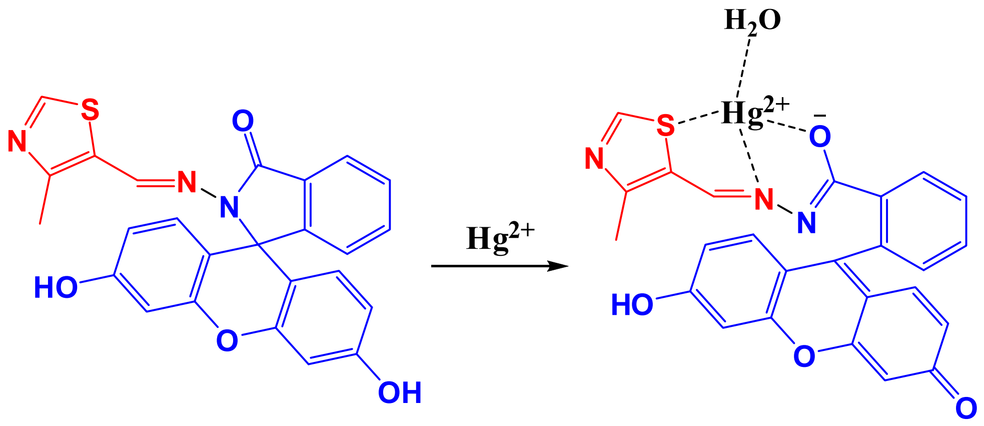

Figure S5. Possible reaction scheme between chemosensor **1** and Hg^{2+} ion

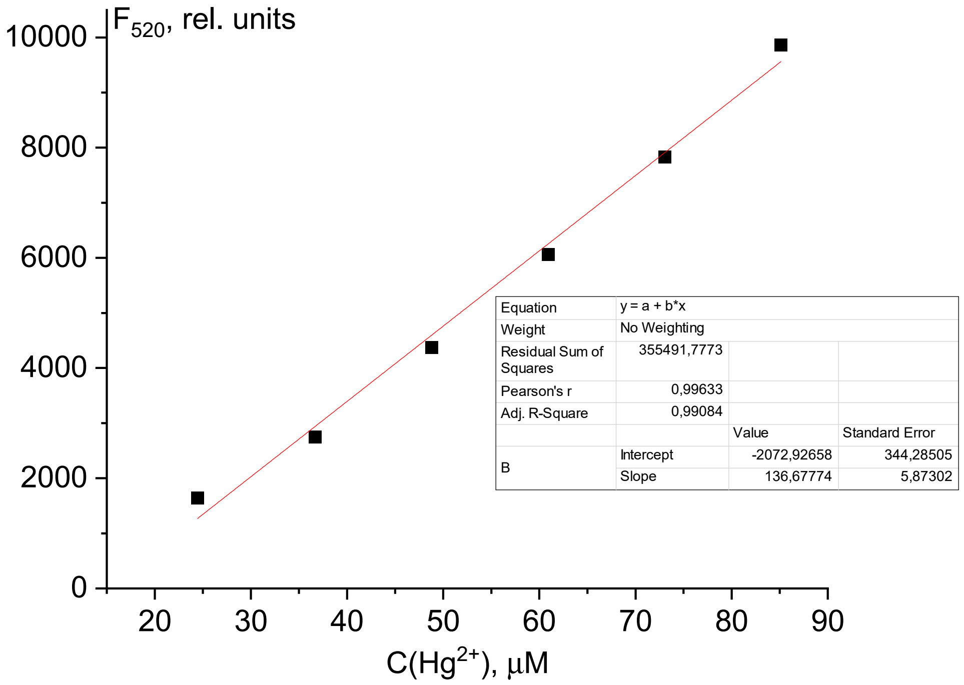

LOD = 3σ/slope = 3*10.6/136.677 = 0.23 μM

Figure S6. The fluorescence detection limit of chemosensor **1** in H_2O-DMSO (8:2 v:v)

Table S1 – Structures of selected fluorescence probes for Hg^{2+} ions in the literature

Ref.	Structure	Signal type	LOD (μM)	Solvent
[1]	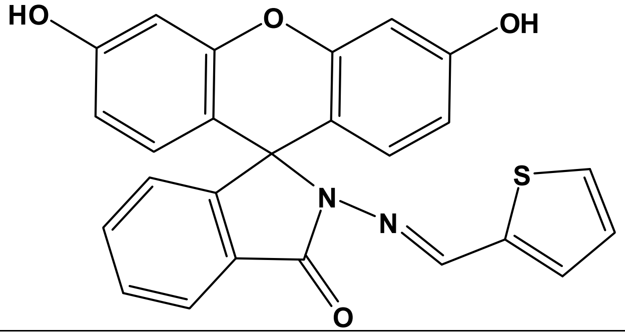	Turn-on	0.24	DMSO/H_2O = 1/9 v/v
[2]	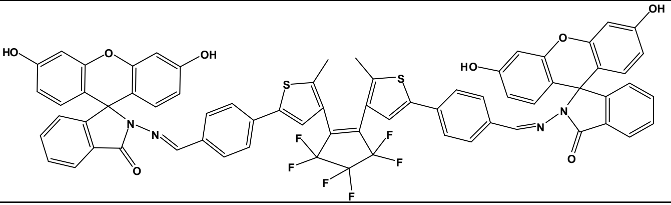	Turn-on	0.33	MeCN
[3]	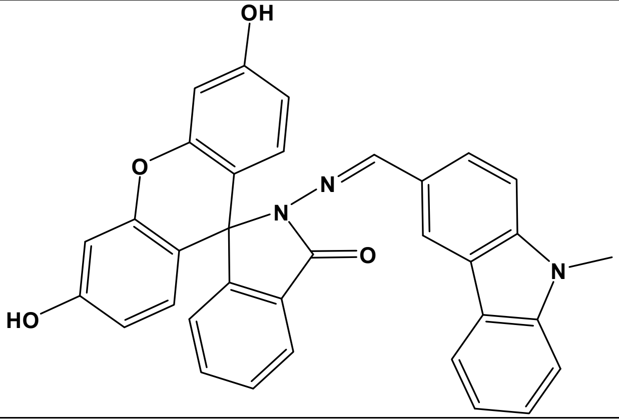	Turn-on	0.04	EtOH/H_2O = 1/1 v/v
[4]	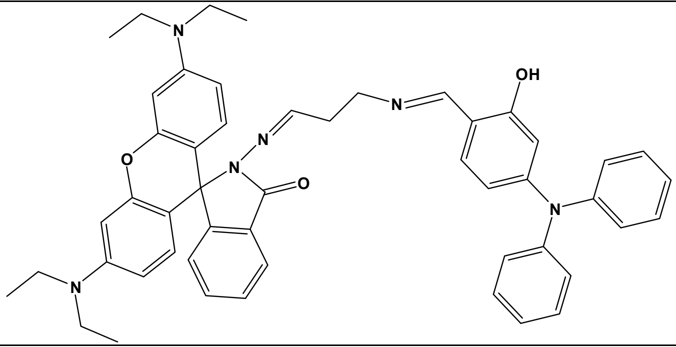	Turn-on	0.48	MeCN/H_2O = 9/1 v/v
[5]	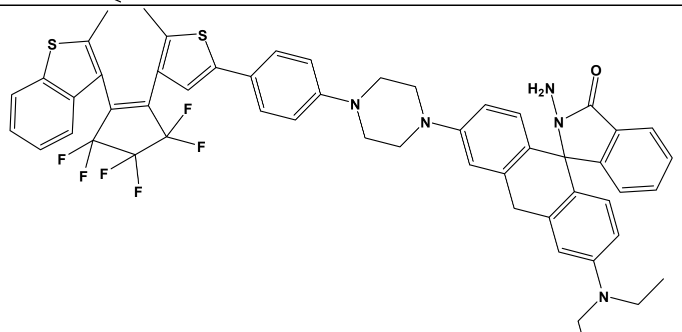	Turn-on	1.16	MeOH/H_2O = 7/3 v/v

[6]	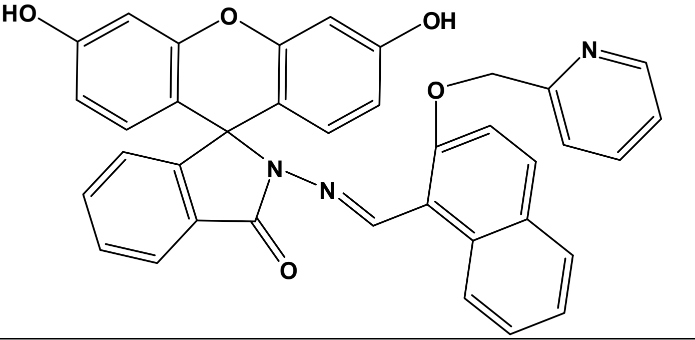	Turn-on	1.24	HEPES buffer, pH 7.2
[7]	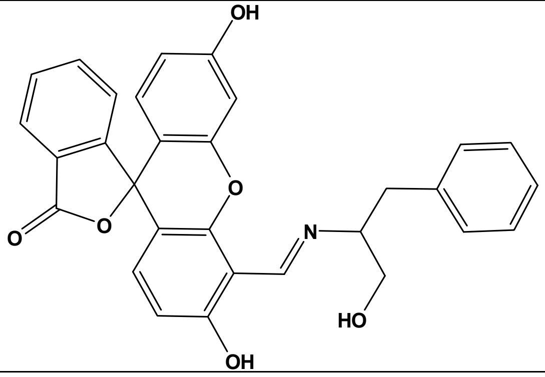	Turn-off	0.34	H_2O
This work	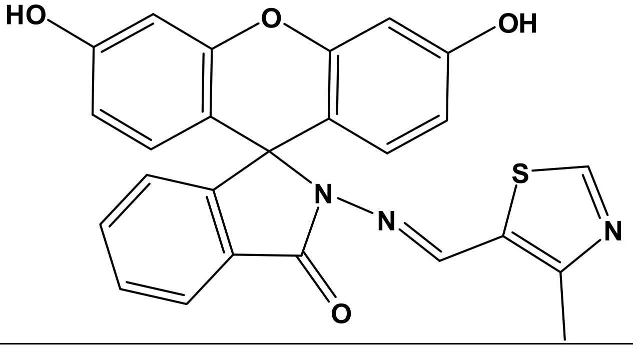	Turn-on	0.23	H_2O/DMSO = 8/2 v/v

References:

1. Chiou, Y.-R.; Wan, C.-F.; Wu, A.-T. A Selective Colorimetric and Turn-on Fluorescent Chemosensor for Hg2+ in Aqueous Solution. *J. Fluoresc.* **2017**, *27*, 317–322, doi:10.1007/s10895-016-1960-7.
2. Kang, H.; Xu, H.; Fan, C.; Liu, G.; Pu, S. A New Sensitive Symmetric Fluorescein-Linked Diarylethene Chemosensor for Hg2+ Detection. *J. Photochem. Photobiol. Chem.* **2018**, *367*, 465–470, doi:10.1016/j.jphotochem.2018.09.001.
3. Huang, L.; Sun, Y.; Zhao, G.; Wang, L.; Meng, X.; Zhou, J.; Duan, H. A Novel Fluorescein-Based Fluorescent Probe for Detection Hg2+ and Bioimaging Applications. *J. Mol. Struct.* **2022**, *1255*, 132427, doi:10.1016/j.molstruc.2022.132427.
4. Erdemir, S. Fluorometric Dual Sensing of Hg2+ and Al3+ by Novel Triphenylamine Appended Rhodamine Derivative in Aqueous Media. *Sens. Actuators B Chem.* **2019**, *290*, 558–564, doi:10.1016/j.snb.2019.04.037.
5. Wang, S.; Ding, H.; Wang, Y.; Fan, C.; Liu, G.; Pu, S. Novel Multi-Responsive Fluorescence Switch for Hg2+ and UV/Vis Lights Based on Diarylethene-Rhodamine Derivative. *Tetrahedron* **2019**, *75*, 1517–1524, doi:10.1016/j.tet.2019.01.071.
6. Mohammad, H.; Islam, A.S.M.; Prodhan, C.; Ali, M. A Fluorescein-Based Chemosensor for “Turn-on” Detection of Hg2+ and the Resultant Complex as a Fluorescent Sensor for S2− in Semi-Aqueous Medium with Cell-Imaging Application: Experimental and Computational Studies. *New J. Chem.* **2019**, *43*, 5297–5307, doi:10.1039/C8NJ05418E.
7. Rathod, R.V.; Bera, S.; Maity, P.; Mondal, D. Mechanochemical Synthesis of a Fluorescein-Based Sensor for the Selective Detection and Removal of Hg $^{2+}$ Ions in Industrial Effluents. *ACS Omega* **2020**, *5*, 4982–4990, doi:10.1021/acsomega.9b03885.

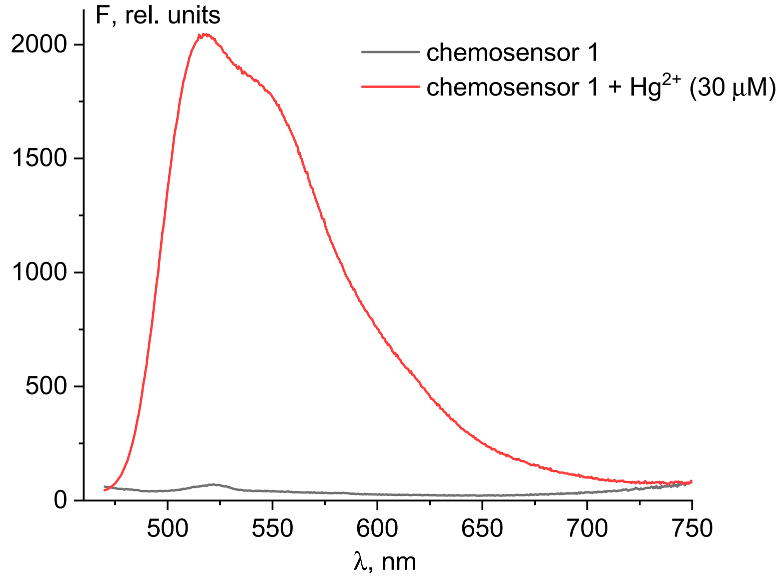

Figure S7. An example of spectral measurements of chemosensor **1** in the presence of Hg^{2+} ions (30 µM) in the sample (Uhtokhma river)

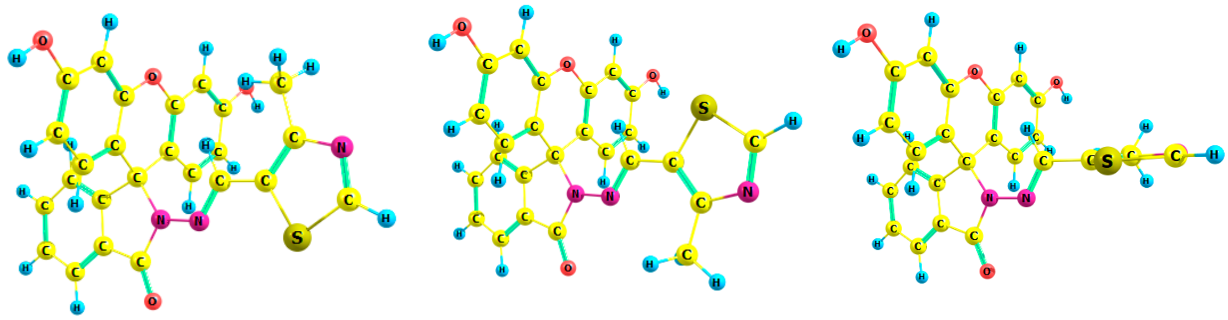

1a	1b	TS
$\Delta E = 0\,\frac{kcal}{mol}$	$\Delta E = 1.55\,\frac{kcal}{mol}$	$\Delta E = 7.7\,\frac{kcal}{mol}$
$\Delta G = 0\,\frac{kcal}{mol}$	$\Delta G = 1.88\,\frac{kcal}{mol}$	$42i$ cm^{-1}
$x = 96\,mol.\%$	$x = 4\,mol.\%$	
**1a**	**1b**	**TS**

Figure S8. Two conformers of **1** and transition state molecular models.

Table S2. Calculated composition of the lowest excited states and corresponding oscillator strengths for **1**.

State	λ, nm	oscillator strength, f[a]	Composition (%)[b]
1 ^{1}A'	319	0,78	7a"→ 1a"* (100)
4 ^{1}A'	257	0,07	5a" →1a"* (5)
			6a" →1a"* (27)
			6a" → 3a"* (5)
			6a" → 4a"* (7)
			5a' →1a'* (45)
6 ^{1}A"	249	0,08	3a'→1a"* (5)
			4a'→1a"* (5)
			4a'→3a"* (10)
			4a'→4a"* (5)
			6a" → 1a'* (27)
			5a' →4a"* (20)
			5a' →5a"* (13)
			7a" → 1a'* (6)
12 ^{1}A"	234	0,05	1a'→1a"* (11)
			2a'→1a"* (6)
			4a'→1a"* (58)
			7a" → 1a'* (19)
15 ^{1}A'	224	0,1	3a" →1a"* (15)
			3a" → 2a"* (8)
			4a" →1a"* (32)
			4a" → 2a"* (6)
			5a" →1a"* (8)
			4a'→1a'* (6)
			6a" → 3a"* (8)
			7a" → 2a"* (7)
16 ^{1}A'	220	0,15	4a" →1a"* (10)
			4a'→1a'* (37)

				6a” → 3a”* (5)
				6a” → 5a”* (10)
				7a” → 3a”* (16)
17 ^{1}A’	218	0,37		4a’→1a’* (25)
				5a’ →1a’* (6)
				7a” → 3a”* (51)
18 ^{1}A”	215	0,12		1a’→1a”* (36)
				4a’→1a”* (7)
				4a’→5a”* (5)
				6a” → 1a’* (16)
				5a’ →2a”* (11)
				5a’ →3a”* (5)
				5a’ →4a”* (8)
20 ^{1}A”	211	0,07		1a’→1a”* (7)
				4a’→5a”* (7)
				6a” → 1a’* (26)
				5a’ →3a”* (12)
				5a’ →4a”* (9)
				7a” → 2a’* (25)
				7a” → 8a’* (5)
21 ^{1}A’	211	0,13		5a” →1a”* (10)
				6a” → 2a”* (11)
				6a” → 3a”* (38)
				5a’ →1a’* (10)
				7a” → 3a”* (20)
22 ^{1}A”	208	0,58		4a’→2a”* (5)
				4a’→3a”* (19)
				6a” → 1a’* (9)
				5a’→3a”* (13)
				5a’ →4a”* (27)

				5a’ →5a”* (11)
23 ^{1}A’	207	0,1		3a” →1a”* (10)
				6a” → 2a”* (29)
				7a” → 4a”* (29)
				7a” → 5a”* (10)
				7a” → 6a”* (22)
28 ^{1}A”	200	0,36		4a’→2a”* (16)
				4a’→3a”* (17)
				4a’→4a”* (6)
				5a’ →5a”* (55)
29 ^{1}A’	198	0,16		2a” →1a”* (17)
				3a” →1a”* (6)
				4a” → 3a”* (5)
				5a” → 3a”* (7)
				6a” → 3a”* (5)
				6a” → 4a”* (44)
				5a’ →1a’* (5)
				7a” → 5a”* (6)
30 ^{1}A’	196	0,08		1a” →1a”* (27)
				2a” →1a”* (5)
				6a” → 4a”* (10)
				7a” → 5a”* (14)
				7a” → 6a”* (35)

[a] bands with f>0,05 presented

[b] transitions with contribution >5% listed

Table S3. Shapes of molecular otbitals participating in electronic transitions

Orbital	Energy, eV	View 1	View 2
HOMO–11<br>1a’	-10.13	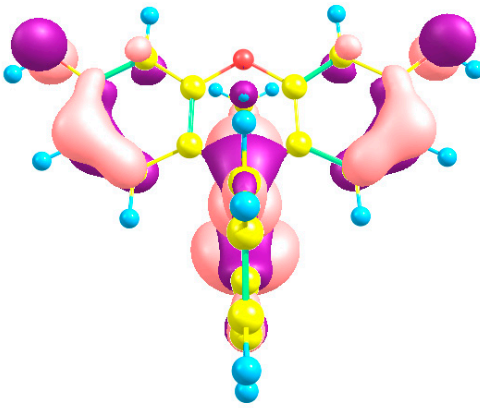	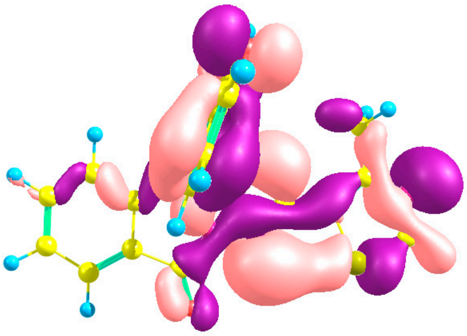
HOMO–10<br>1a”	-10.06	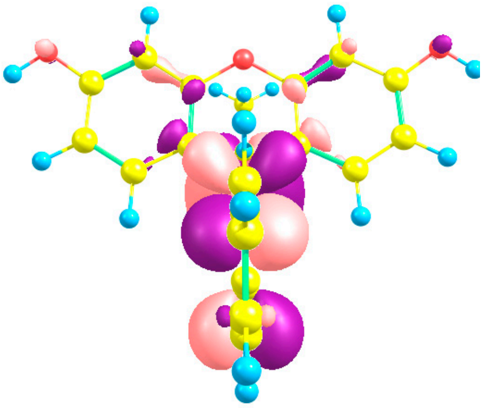	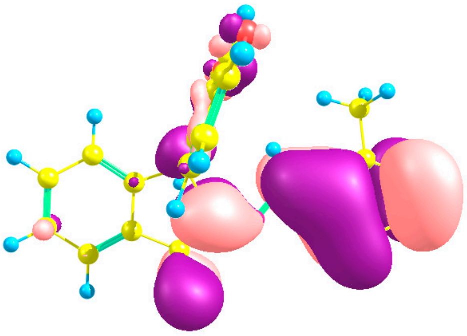
HOMO–9<br>2a’	-9.64	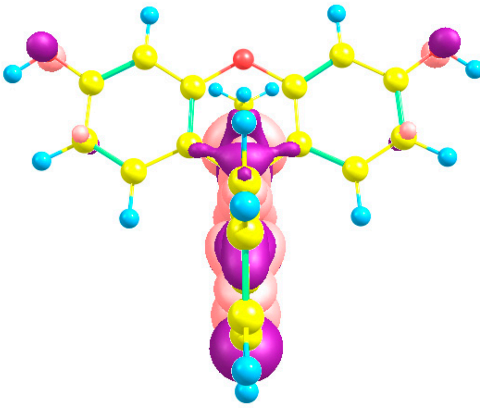	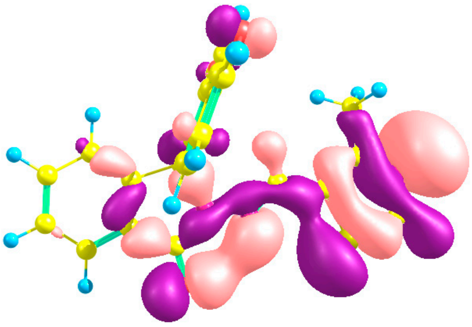
HOMO–8<br>3a’	-9.54	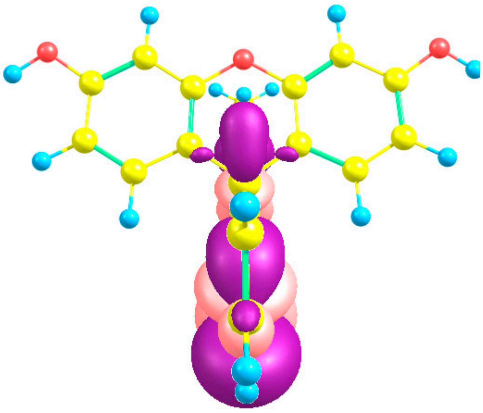	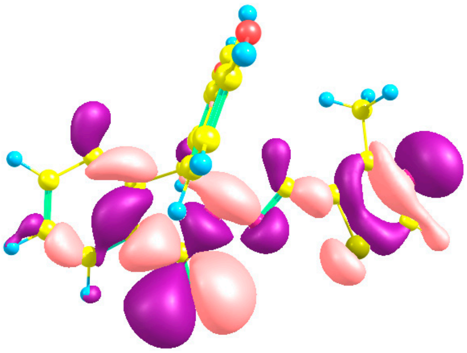

HOMO–7<br>2a”	-9.25	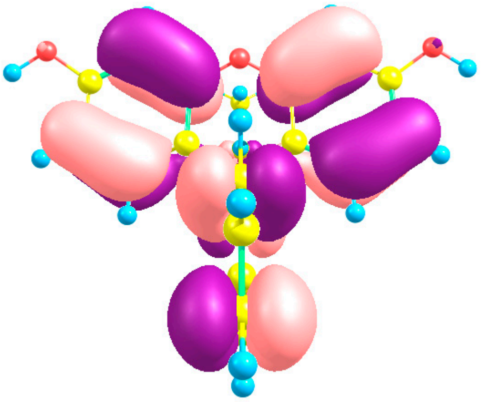	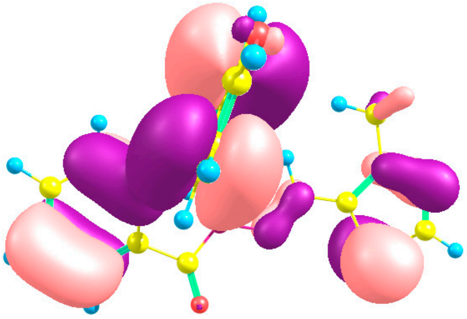
HOMO–6<br>3a”	-9.16	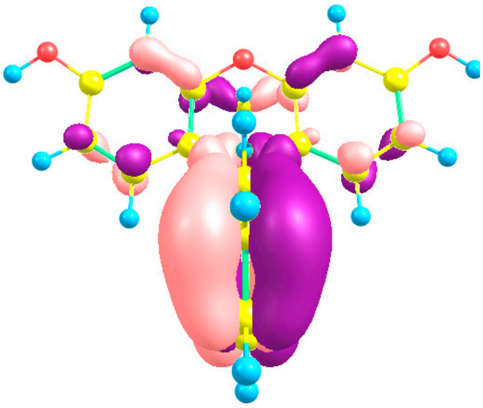	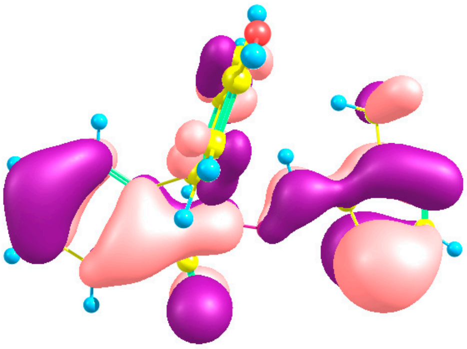
HOMO–5<br>4a”	-8.99	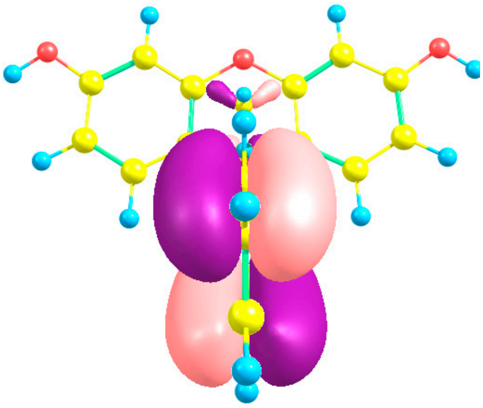	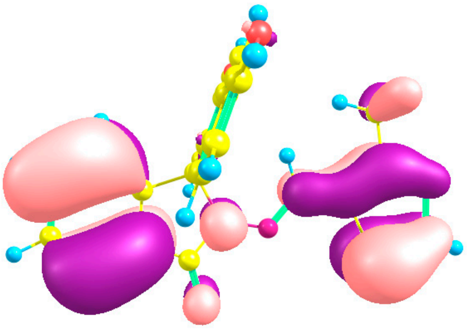
HOMO–4<br>5a”	-8.94	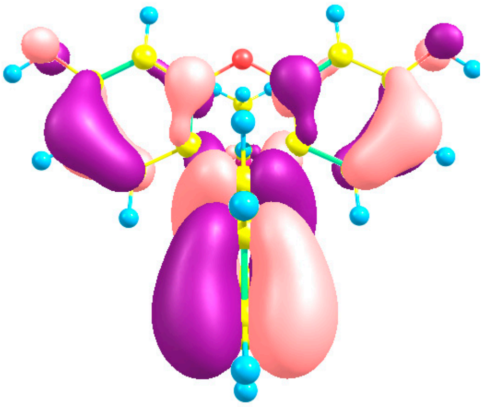	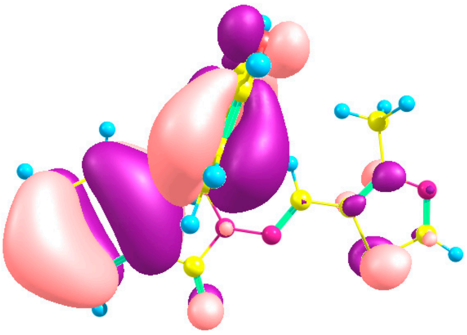

HOMO–3<br>4a’	-8.32	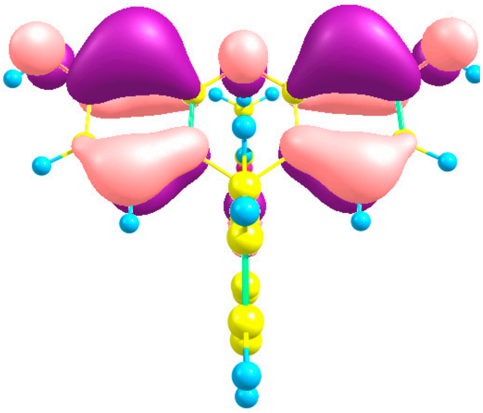	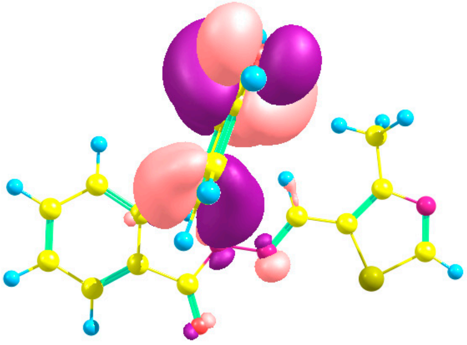
HOMO–2<br>6a”	-8.05	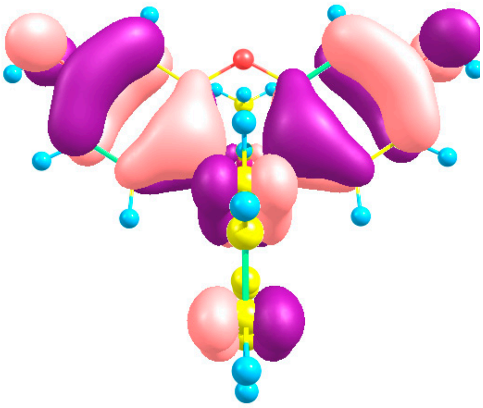	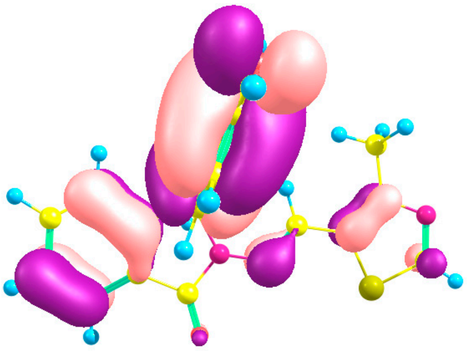
HOMO–1<br>5a’	-7.74	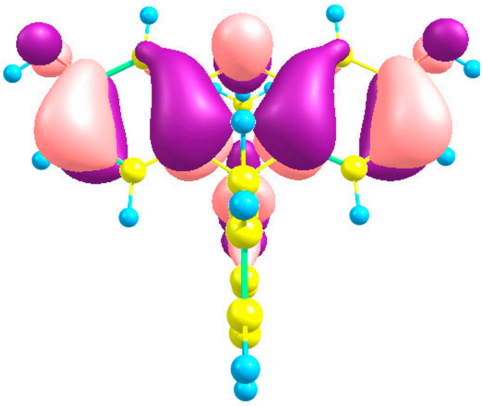	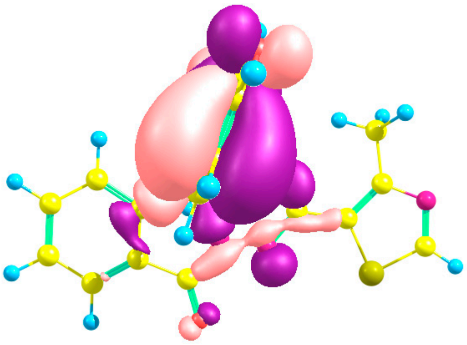
HOMO<br>7a”	-7.55	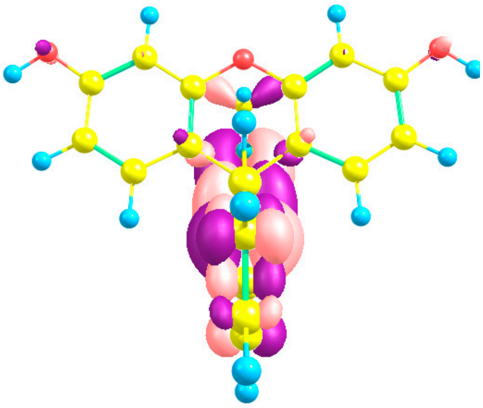	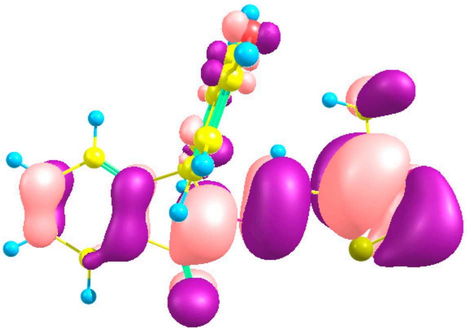

LUMO<br>1a"*	-1.14	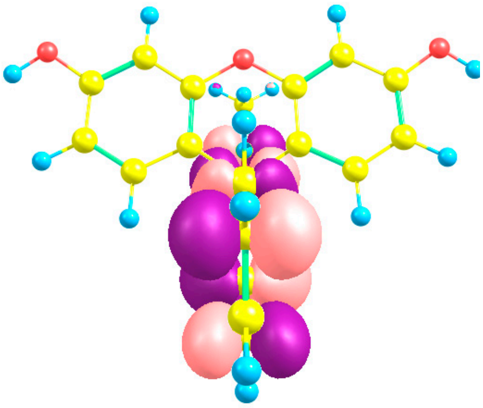	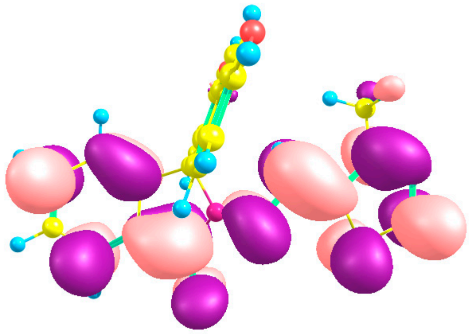
LUMO+1<br>2a"*	-0.18	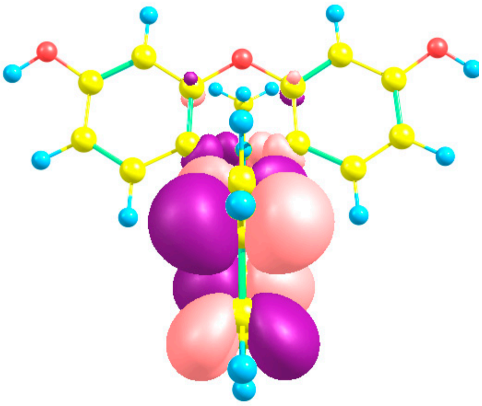	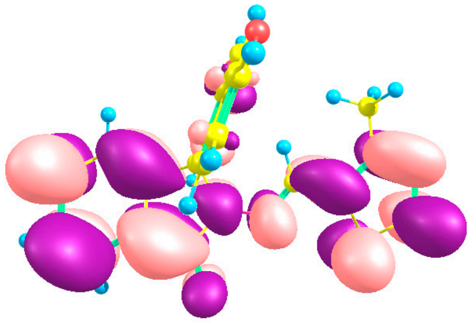
LUMO+2<br>1a'*	0.01	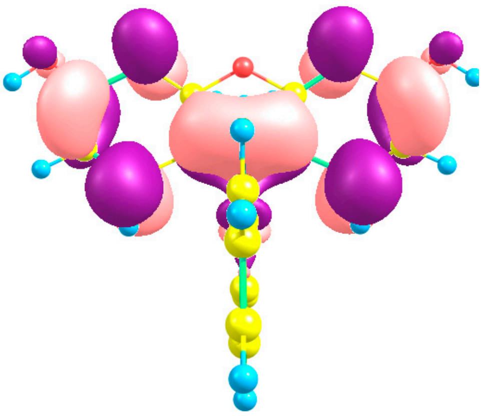	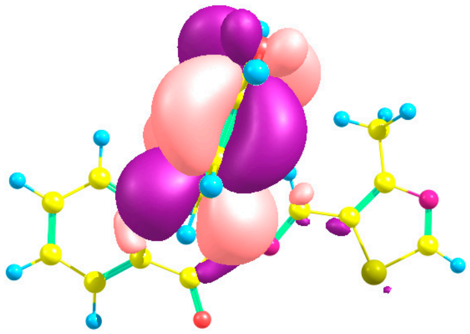
LUMO+3<br>3a"*	0.13	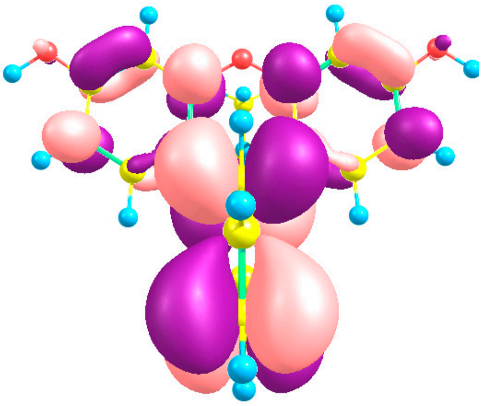	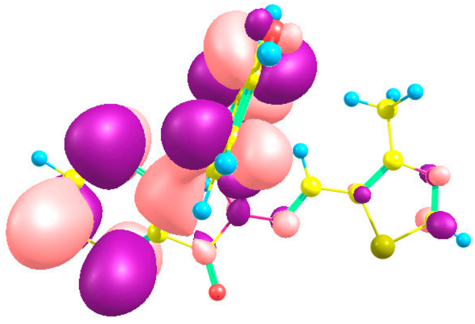

LUMO+4<br>4a”*	0.53	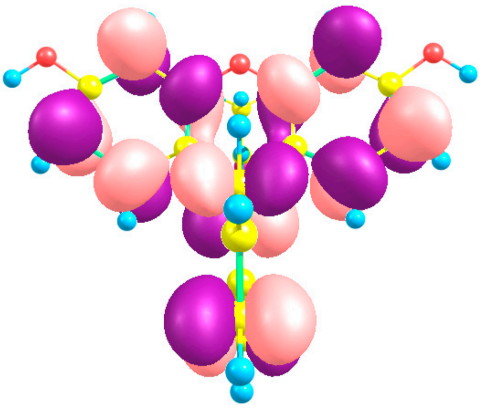	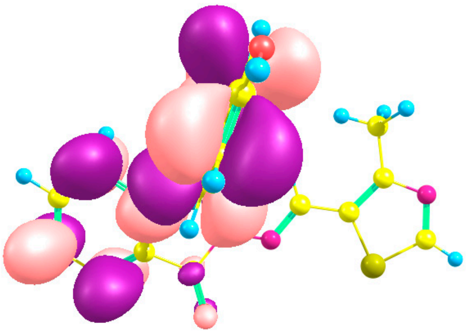
LUMO+5<br>5a”*	0.76	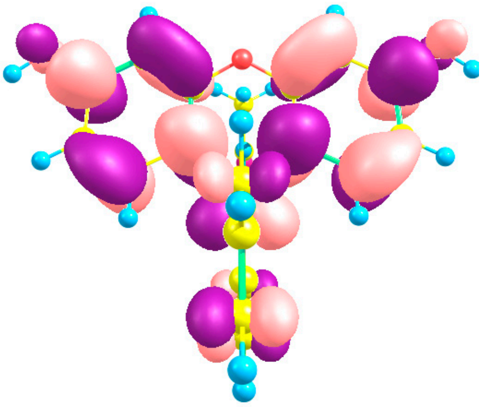	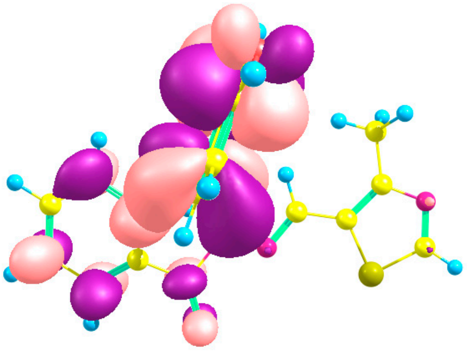
LUMO+6<br>2a’*	1.02	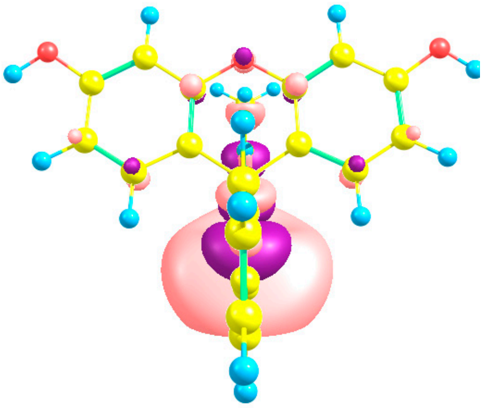	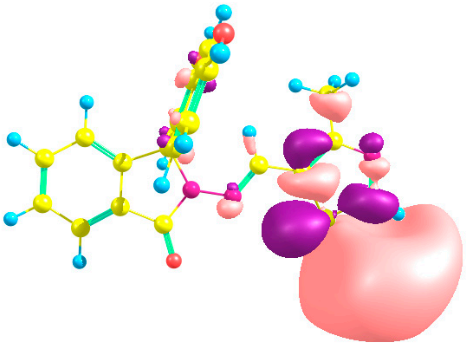
LUMO+7<br>6a”*	1.21	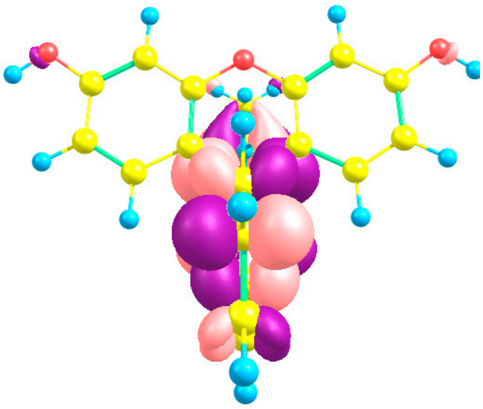	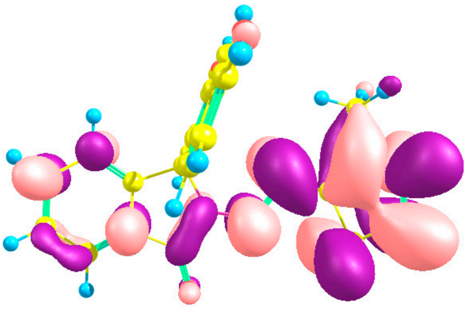

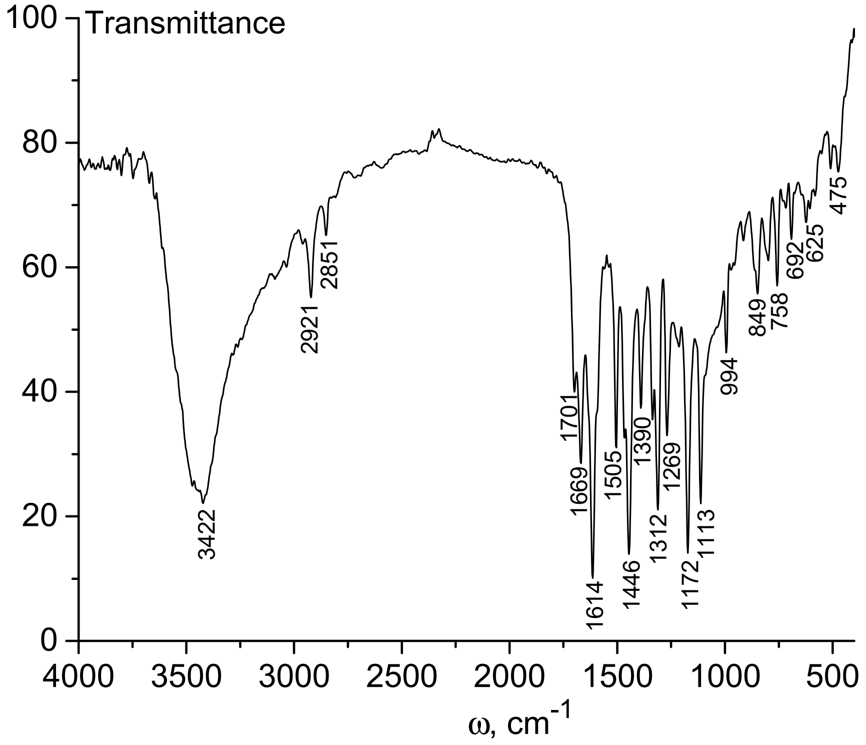

Figure S9. IR spectrum of chemosensor **1**

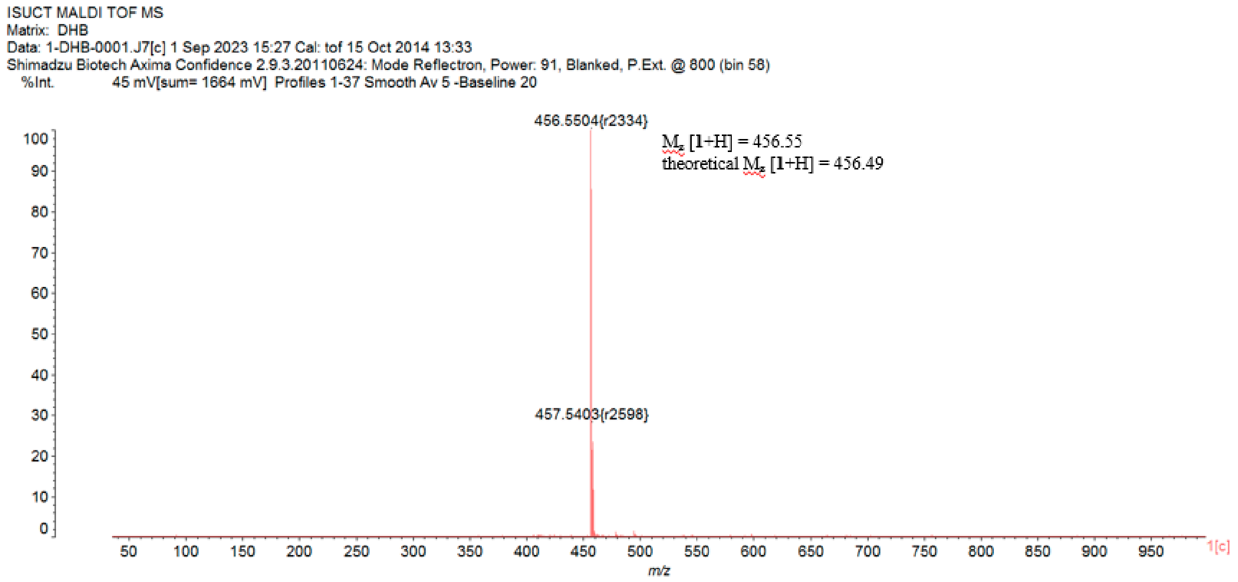

Figure S10. MALDI TOF mass spectrum of chemosensor **1**

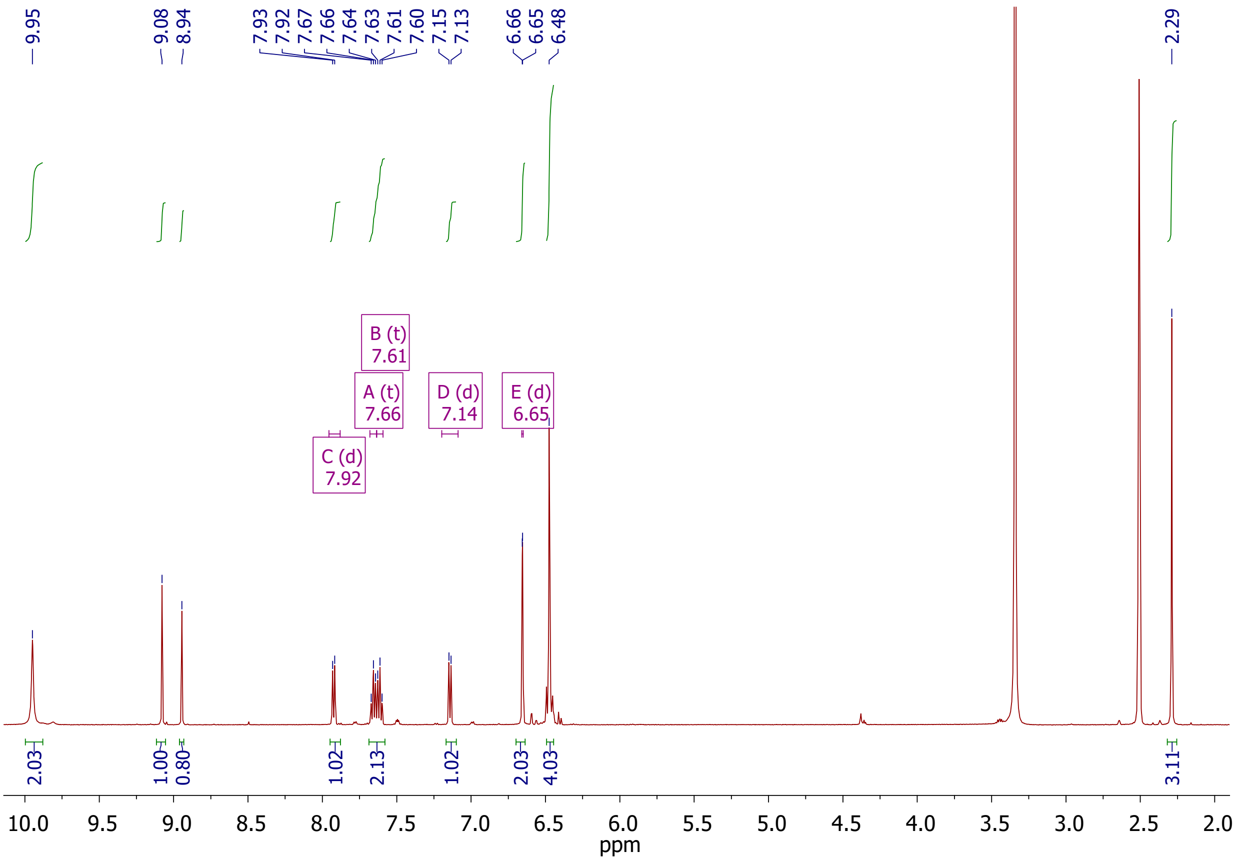

Figure S11. ^{1}H NMR spectra of chemosensor **1**

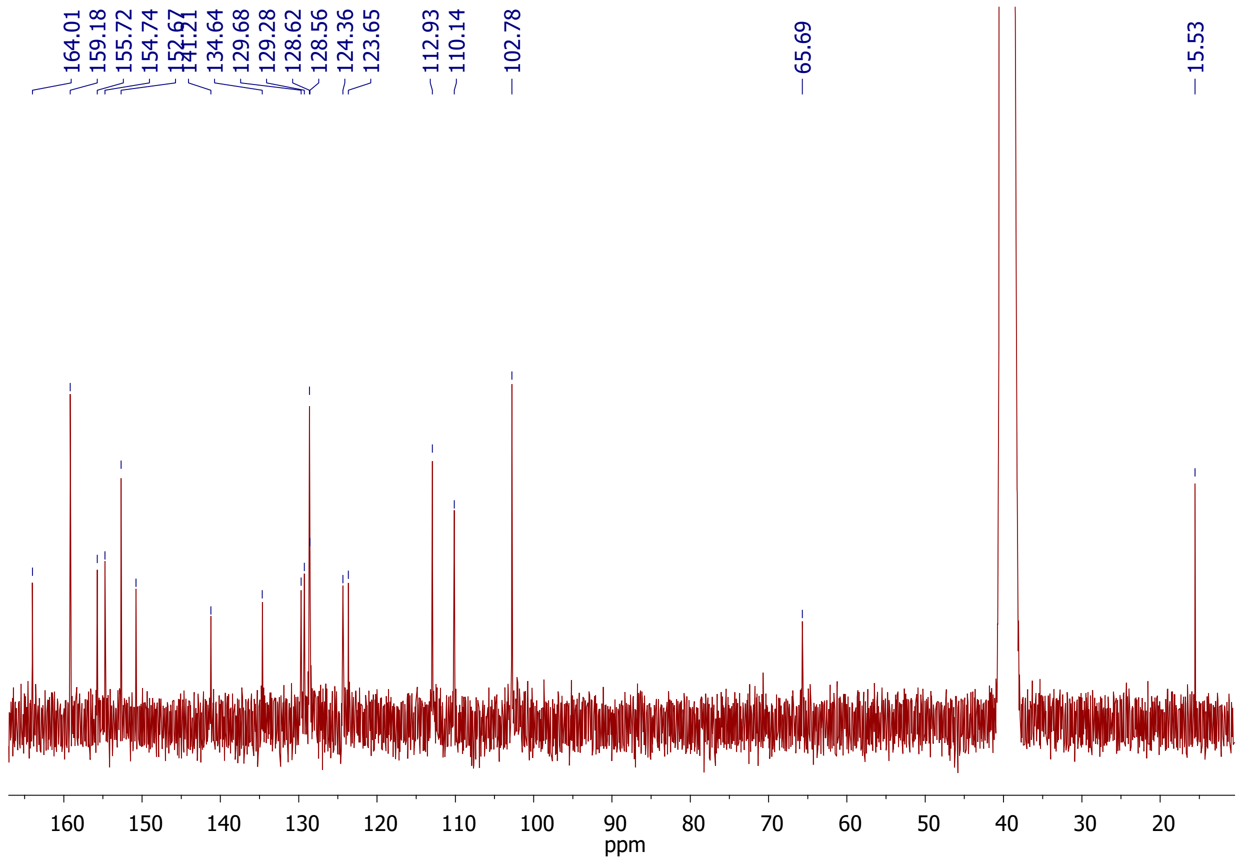

Figure S12. ^{13}C NMR spectra of chemosensor **1**

Cartesian coordinates of 1a, calculated at B97D/def2-TZVP level of theory

C	0.136193000	-2.339592000	-2.674602000
C	0.425991000	-1.189189000	-1.937473000
O	0.237681000	-0.000162000	-2.607670000
C	0.857476000	-1.253392000	-0.606964000
C	0.857579000	1.253295000	-0.607160000
C	0.425971000	1.188938000	-1.937598000
C	0.275001000	-3.587906000	-2.068184000
C	0.986903000	-2.519875000	-0.024903000
C	0.987166000	2.519844000	-0.025271000
C	0.136055000	2.339260000	-2.674820000
C	0.275004000	3.587645000	-2.068574000
C	0.701140000	3.681260000	-0.732208000
H	1.313065000	2.584843000	1.010121000
H	-0.202280000	2.257448000	-3.703217000
H	0.803387000	4.656327000	-0.258547000
C	0.700900000	-3.681361000	-0.731732000
H	-0.202019000	-2.257888000	-3.703049000
H	1.312666000	-2.584803000	1.010539000
H	0.803021000	-4.656372000	-0.257925000
O	-0.022622000	-4.688302000	-2.824382000
H	0.103893000	-5.483394000	-2.287700000
O	-0.022743000	4.687960000	-2.824847000
H	0.104102000	5.483119000	-2.288340000
C	3.778371000	-0.000092000	-0.155204000
C	2.636836000	-0.000025000	0.638662000
C	2.725838000	0.000088000	2.027123000
C	3.962031000	0.000143000	2.676092000
C	5.114843000	0.000071000	1.887830000
C	5.022589000	-0.000049000	0.485340000
C	1.185141000	0.000001000	0.184746000
N	0.485336000	0.000143000	1.520922000
C	1.371329000	0.000180000	2.625898000
H	3.705885000	-0.000170000	-1.240679000
H	4.006849000	0.000249000	3.762598000
H	6.095006000	0.000102000	2.359526000
H	5.932914000	-0.000100000	-0.110451000
O	1.056403000	0.000262000	3.798185000
N	-0.837164000	0.000220000	1.742779000
C	-1.669618000	0.000178000	0.751274000
C	-3.092430000	0.000184000	0.970039000
C	-4.089183000	-0.000038000	-0.000410000
N	-5.374057000	-0.000171000	0.485244000
C	-5.377344000	-0.000135000	1.792326000
S	-3.820944000	0.000261000	2.551675000
H	-6.284998000	-0.000197000	2.389966000

Cartesian coordinates of 1b, calculated at B97D/def2-TZVP level of theory

C	-0.425796000	2.401104000	-2.699649000
C	-0.631167000	1.233393000	-1.961110000
O	-0.539090000	0.061286000	-2.678382000

C	-0.897018000	1.266491000	-0.586638000
C	-0.891497000	-1.239370000	-0.643889000
C	-0.624993000	-1.142618000	-2.015187000
C	-0.480631000	3.634485000	-2.050570000
C	-0.944724000	2.518068000	0.038036000
C	-0.933649000	-2.518255000	-0.076807000
C	-0.413309000	-2.274536000	-2.805683000
C	-0.462291000	-3.536346000	-2.213288000
C	-0.720662000	-3.661685000	-0.837180000
H	-1.131983000	-2.608607000	0.988605000
H	-0.203018000	-2.167891000	-3.865483000
H	-0.752732000	-4.646985000	-0.374805000
C	-0.737949000	3.695832000	-0.669850000
H	-0.215921000	2.343699000	-3.763327000
H	-1.142698000	2.558775000	1.106556000
H	-0.774281000	4.658934000	-0.163116000
O	-0.270851000	4.752621000	-2.810363000
H	-0.323654000	5.534028000	-2.242170000
O	-0.245871000	-4.617572000	-3.023027000
H	-0.293218000	-5.424350000	-2.490954000
C	-3.763035000	-0.009644000	0.082628000
C	-2.558438000	-0.022029000	0.776837000
C	-2.530076000	-0.053152000	2.167729000
C	-3.706866000	-0.072794000	2.918887000
C	-4.922168000	-0.060484000	2.231094000
C	-4.948440000	-0.029227000	0.826077000
C	-1.149829000	-0.005681000	0.202343000
N	-0.340680000	-0.033049000	1.472566000
C	-1.129861000	-0.061235000	2.647938000
H	-3.782562000	0.014232000	-1.004811000
H	-3.659313000	-0.096963000	4.004992000
H	-5.859028000	-0.075280000	2.783561000
H	-5.905872000	-0.020225000	0.309412000
O	-0.713063000	-0.086536000	3.788605000
N	0.993585000	-0.033254000	1.590833000
C	1.753865000	-0.008247000	0.542164000
C	3.191155000	-0.010388000	0.685332000
C	3.994967000	-0.043809000	1.826412000
N	5.342755000	-0.035373000	1.576917000
C	5.596631000	0.003088000	0.293988000
S	4.203055000	0.032518000	-0.729066000
H	6.597840000	0.016449000	-0.126552000
C	3.494945000	-0.086339000	3.237654000
H	4.345788000	-0.128341000	3.924911000
H	2.875657000	0.792154000	3.460016000
H	2.838492000	-0.951437000	3.394675000
H	1.364021000	0.015133000	-0.476953000

Figure S13. Cartesian coordinates of quantum chemical structures
